# Disentangling Auger decays in O_2_ by photoelectron-ion coincidences

**DOI:** 10.1038/s41598-017-02875-x

**Published:** 2017-06-06

**Authors:** Xiao-Jing Liu, Christophe Nicolas, Minna Patanen, Catalin Miron

**Affiliations:** 1 School of Physics and Nuclear Energy Engineering, Beihang University, Beijing, 100191 China; 2 Synchrotron SOLEIL, l’Orme des Merisiers, Saint-Aubin, BP 48, 91192 Gif-sur-Yvette Cedex, France; 3 Nano and Molecular Systems Research Unit, Molecular Materials Research Community, Faculty of Science, University of Oulu, P.O. Box 3000, 90014 Oulu, Finland; 4 Extreme Light Infrastructure Nuclear Physics (ELI-NP), “Horia Hulubei” National Institute for Physics and Nuclear Engineering, 30 Reactorului Street, RO-077125 Măgurele, Jud. Ilfov Romania

## Abstract

In non-resonant Auger electron spectroscopies, multi core-ionized states lead to numerous energetically close-lying electronic transitions in Auger spectra, this hampering the assignment and interpretation of the experimental results. Here we reveal a new method to overcome this intrinsic limitation of non-resonant inner-shell spectroscopies. In a proof-of-principle experiment performed for the O_2_ molecule, most of the Auger final states are dissociative, and we measure in coincidence the kinetic energy of the photoelectron and the kinetic energy release of the (O^+^, O^+^) ion pairs produced after the Auger decay of the O 1s^−1^ core-ionized states. The Auger final states are assigned using energy conservation. We fully separate the contributions from the ^4^Σ^−^ and ^2^Σ^−^ intermediate ionic states and conclusively demonstrate that the Auger decay probability can dramatically depend on the different O_2_ 1*s* ^−1^ intermediate multiplet states. In addition, a metastable Auger final state also exists, with lifetime longer than 3.8 *μ*s, and clear changes are observed in both branching ratio and spectral profile of the O 1s photoelectron spectrum when they are recorded in coincidence with either ${{\bf{O}}}_{{\bf{2}}}^{{\boldsymbol{+}}{\boldsymbol{+}}}$ or with other ionic species. These changes are attributed to the population of the metastable ${{\boldsymbol{B}}}^{{\boldsymbol{^{\prime} }}3}{{\boldsymbol{\Sigma }}}_{{\boldsymbol{u}}}^{-}({\boldsymbol{\nu }}{\boldsymbol{^{\prime\prime} }}{\boldsymbol{=}}0)$ Auger final state via different intermediate states.

## Introduction

Developed by Siegbahn in the 1950’s^1^, the electron spectroscopy for chemical analysis (ESCA) is widely used in characterizing the properties of atoms, molecules and condensed matter. This technique has emerged from fundamental science and evolved as a useful analytical tool for materials research. Non-resonant inner-shell spectroscopies, and in particular X-ray photoelectron spectroscopy (XPS) and Auger electron spectroscopy (AES) are able to provide information not only on the elemental composition, but also on the chemical state of the elements in materials. When a material is shined with electromagnetic radiation of sufficiently high photon energy, X-ray emission or Auger decay occur after an electron is removed from a core-orbital through photoionization. While in the first approximation XPS provides one with the one-particle density of states, and further restricting the discussion to the non-radiative decay, the AES carries information about the two-particle density of states, based on which one can obtain the hole-hole Coulomb interactions^2, 3^. However, especially in molecules, several factors limit the use of conventional AES. First, there exist many different, close-lying intermediate states: vibrational sub-states, spin-orbit split, shake-up or shake-off components of core-hole states, or core-hole states from elements of the same Z but with different chemical shifts^2^. Second, there is a large number of dicationic final states involved in the Auger decay^4–7^. Third, depending on its potential energy surface, each Auger final state can be bound, predissociative or dissociative. The interplay between nuclear motion and electronic decay will play a role, strongly affect the profile of the Auger lines, and make the spectral analysis rather complex^8, 9^. As a result, Auger spectra are often subject to spectral congestion with overlapping peaks of various shapes^10, 11^.

Electron-electron coincidence spectroscopy offers the opportunity of disentangling the contributions from individual intermediate states to the Auger spectrum and allows for a better separation of the final states, which otherwise are superimposed in conventional spectroscopic techniques. The first experiment was pioneered on copper by Haak *et al*.^12^ in the late seventies. Recent works on bulk and adsorbate systems were reviewed by Stefani *et al*.^13^. This technique was first introduced for isolated species by Kämmerling and Schmidt^14^. Later, using a multi-electron TOF spectrometer setup, Viefhaus *et al*. showed interference between the photoelectron emission and the Auger electron emission in atomic Xe^15^. Coupling a high resolution electrostatic analyzer with a number of electron time-of-flight spectrometers, Ulrich *et al*. separated the decay from selected vibrational sub-states of the C 1s^−1^ core-hole intermediate state in the Auger decay channels of the CO molecule^16^. Bolognesi *et al*. performed a similar measurement by using several energy dispersive electron analyzers^17^. In order to improve the detection efficiency while keeping good energy resolution, an ArTOF spectrometer^18^ was used by Hergenhahn’s group^19^. The ultimate effort to improve the detection efficiency ended up with the use of a magnetic bottle spectrometer^20^ to perform multi-electron coincidence measurements^21, 22^. With it, the acceptance angle approached 4*π* sr with a typical energy resolution ${\rm{\Delta }}E/E\approx \mathrm{1.6 \% }$, but the drawbacks of this technique are that the electron emission angles cannot be recorded, and the energy resolution is not sufficient to resolve the spin-orbit split Auger final states. By using a Cold Target Recoil Ion Momentum Spectrometer COLTRIMS^23, 24^, normally coincidence measurements are performed for electron kinetic energies below a few tens of eV with a resolution ${\rm{\Delta }}E/E\approx \mathrm{5 \% }$. However, in this case, the post-collision interaction effect will play a significant role and the situation becomes more complicated. Using a retarding electric field, electron kinetic energy can be extended to several hundred eV with expense of detection efficiency^25^. The efforts are being pursued to further improve the resolution and the detection efficiency of such measurements.

O_2_ molecule has been often selected as a testbed for experiments and theoretical predictions. Sorensen *et al*.^26^ reported a high resolution O 1s photoelectron spectrum of O_2_, in which the energy splitting due to exchange coupling, molecular vibration and the *gerade*/*ungerade* symmetries were resolved. Early studies focusing on the normal Auger spectrum of O_2_ were performed by Siegbahn *et al*.^2^ and Moddeman *et al*.^27^. Later, Larsson *et al*. described in detail the Auger spectrum of O_2_ by combined high-resolution electron spectroscopy and CASSCF/MRCI calculations^28^. This work was followed by Sorensen *et al*. with improved resolution using synchrotron radiation at SPring-8^29^. Larsson’s experimental data were reanalyzed by Bao *et al*. with the help of extended MRCI calculations and spectral fitting to find the most suitable potential energy curves for both intermediate ionic states and Auger final states^30^. Most recently, Ulrich *et al*.^31^ and Arion *et al*.^19^ separated the contributions to the Auger decays from the two core-hole intermediate states (^4^Σ^−^ and ^2^Σ^−^) using photoelectron-Auger electron coincidences, but the statistical quality of the data was limited due to the reduced electron collection efficiency achieved in these electron-electron coincidence measurements.

An alternative way of probing the electronic states of ${{\rm{O}}}_{2}^{++}$ dication is the Doppler free kinetic energy release spectroscopy by Lundqvist *et al*.^32^. They observed series of vibrational states in the spectrum, which were explained as being the result of the dissociation of metastable dicationic states by tunneling or predissociation through spin-orbit interaction with another dissociative state. On the other hand, the high intensity broad peaks observed in the spectra were not discussed, because all singlet, triplet and quintet states are allowed, thus being all produced in the electron-impact ionization processes, overlapping in the spectrum without well defined line-shapes.

Here, we report on a new method to investigate the Auger decays subsequent to O 1s photoionization in O_2_. By exploring the correlation between the photoelectron kinetic energy and the kinetic energy release of the O^+^/O^+^ ion pair, we disentangle the Auger decay processes from the various intermediate singly ionized states. By measuring in coincidence the photoelectrons and the stable dications ${{\rm{O}}}_{2}^{++}$, the very weak Auger decay channel to the metastable ${B}^{^{\prime} 3}{{\rm{\Sigma }}}_{u}^{-}$ state is revealed.

## Results and Discussion

**Figure 1:**
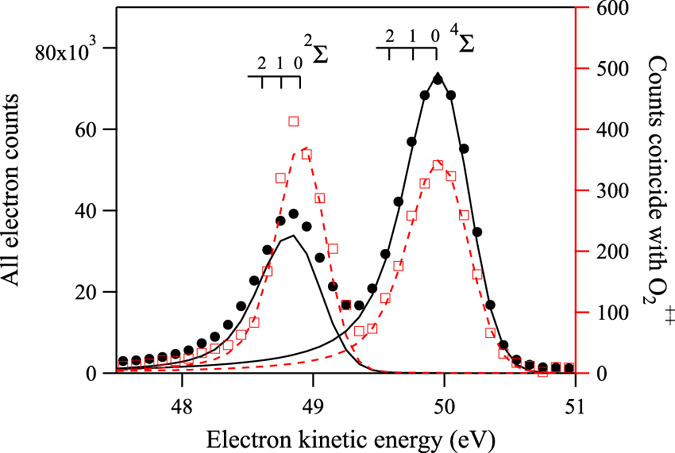
O 1s^−1^ electron energy spectra of O_2_ recorded at 593.4 eV photon energy. The total experimental electron energy spectrum is represented by solid circles and it is fitted by solid lines. The partial electron energy spectrum constructed from photoelectrons arriving in coincidence with ${{\rm{O}}}_{2}^{++}$ is represented by empty squares and it is fitted by dashed lines. Two main states, $1{s}^{-1}({}^{2}{{\rm{\Sigma }}}^{-})$ and $1{s}^{-1}({}^{4}{{\rm{\Sigma }}}^{-})$, are well resolved. The intensity of $1{s}^{-1}({}^{2}{{\rm{\Sigma }}}^{-})$ line is greatly enhanced with respect to $1{s}^{-1}({}^{4}{{\rm{\Sigma }}}^{-})$ line when photoelectrons are recorded in coincidence with ${{\rm{O}}}_{2}^{++}$ ions. The vibrational levels are adopted from ref. 26.

The electron energy spectrum is shown in Fig. 1. At such electron kinetic energy, the post collision interaction effect can be neglected. According to the latest published high-resolution measurement of the O 1s photoelectron spectrum of O_2_ by Sorensen^26^, the energy separation of the 1*s* ^−1^ (^4^Σ^−^) and 1*s* ^−1^ (^2^Σ^−^) lines is 1.04 eV. The vibrational frequencies for the ^4^Σ^−^ and the ^2^Σ^−^ are 180 meV and 145 meV, respectively, and the *gerade*/*ungerade* splitting for the ^4^Σ^−^ and the ^2^Σ^−^ are 50 meV and 7 meV, respectively. Limited by the lifetime broadening of 140 meV^26^ and our present energy resolution of 400 meV, we can only resolve the two main components 1*s* ^−1^ (^4^Σ^−^) and 1*s* ^−1^ (^2^Σ^−^). By least square fitting of the spectrum with two asymmetric Voigt profiles^33^, the value of the ratio $[1{s}^{-1}({}^{4}{{\rm{\Sigma }}}^{-})]/[1{s}^{-1}({}^{2}{{\rm{\Sigma }}}^{-})]$ is determined to be 2.18(2). It agrees quite well with the value of 2.15(5) reported by Sorensen^26^, but is marginally smaller than that of 2.5 reported by Siegbahn^2^ and Bao^30^. However, this value of the ratio is slightly larger than the statistical ratio of 2.0, which means that the multi-electron transitions (satellites) associated with the ^2^Σ^−^ state reduce the intensity of the main line more significantly than the ones associated with the ^4^Σ^−^ state.

**Figure 2:**
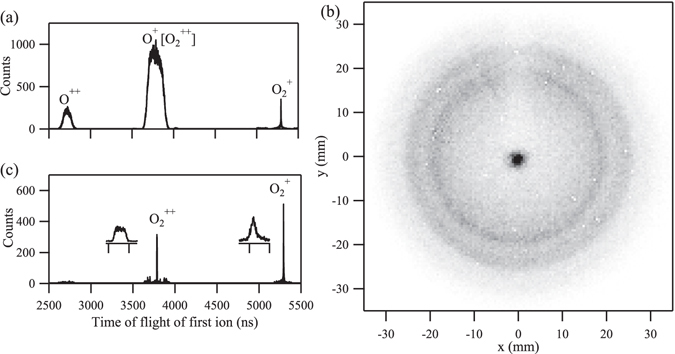
The ion time-of-flight spectrum (**a**) contains a sharp peak corresponding to the parent molecular ion, and two broad peaks corresponding to fragment atomic ions. In the ion image (**b**), the parent molecular ions arrive at the center, while the fragment atomic ions form concentric rings. The shadow observed in the upper-half is due to the gas needle. The selected time-of-flight spectrum corresponding only to ions arriving at center of the detector is shown in panel (c). Expanded views of parent ions are shown in the inserted plot in panel (c).

The ion time-of-flight spectrum is shown in Fig. 2(a). Besides the charged atomic fragments, the singly charged parent molecule is also observed. ${{\rm{O}}}_{2}^{+}$ contributes to 0.89% of the total ion yield, corresponding to the portion of core-ionized ${{\rm{O}}}_{2}^{+}$ that undergoes a radiative decay. This value agrees well with that of 0.83% for the oxygen atom^34^. The peaks associated to the charged atomic fragments are much broader than that of the parent molecular ion, because the former gain kinetic energy during the fragmentation, while the latter does not. The ion detector image is shown in Fig. 2(b). In the image, the concentric rings and a central peak correspond to the atomic fragment ions and the parent molecular ion, respectively. The trench observed in the upper-half is due to the ions blocked by the gas needle. By selecting the center peak in the ion image, the energetic fragments are suppressed, and a sharp peak of ${{\rm{O}}}_{2}^{++}$ appears in the ion time-of-flight spectrum, which accounts for 0.85% of the total ion yield, as shown in Fig. 2(c). In the inserted plots, the peak of ${{\rm{O}}}_{2}^{++}$ has a trapezoid shape with a width of 5.8 ns, compared with that of ${{\rm{O}}}_{2}^{+}$ having a Gaussian shape with a width of 2.8 ns. This is because, compared with ${{\rm{O}}}_{2}^{+}$, ${{\rm{O}}}_{2}^{++}$ receives additional recoil momentum during Auger electron emission. We plot the photoelectron energy spectrum recorded in coincidence with ${{\rm{O}}}_{2}^{++}$ in Fig. 1. The values of the $[1{s}^{-1}({}^{4}{{\rm{\Sigma }}}^{-})]/[1{s}^{-1}({}^{2}{{\rm{\Sigma }}}^{-})]$ ratio when the electron spectrum is measured in coincidence with various ions are summarized in Table 1. It can be readily seen that this ratio is significantly lower in coincidence with ${{\rm{O}}}_{2}^{++}$, in comparison with the other ionic species.

**Table 1:** Ratio between spin-orbit split components of the core-ionized state $1{s}^{-1}({}^{4}{{\rm{\Sigma }}}^{-})/1{s}^{-1}({}^{2}{{\rm{\Sigma }}}^{-})$.

Present measurements					Ref. 30	Ref. 2	Ref. 26
NC^1^	${{\bf{O}}}_{{\bf{2}}}^{{\boldsymbol{+}}}$	O^+^	O^++^	${{\bf{O}}}_{{\bf{2}}}^{{\boldsymbol{+}}{\boldsymbol{+}}}$			
2.18(3)	2.5(2)	2.17(3)	2.29(8)	1.2(1)	2.5	2.5	2.15(5)

**Figure 3:**
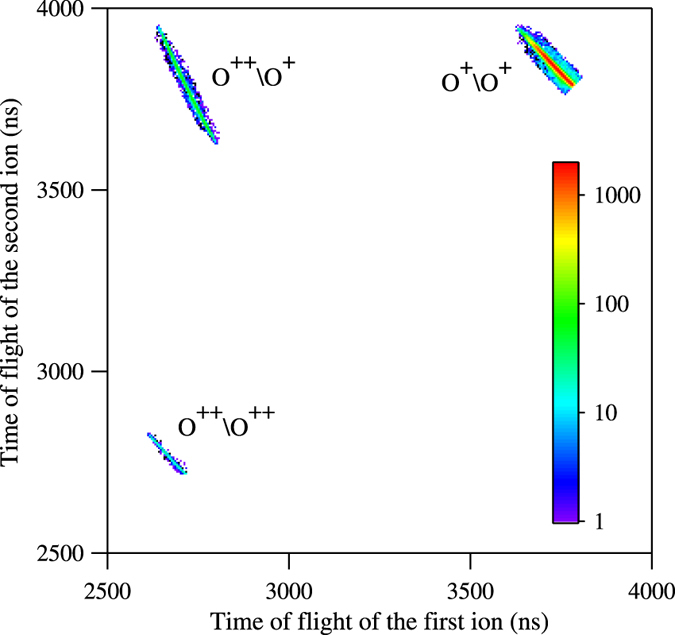
Photoion-photoion coincidence map (PIPICO) represented on a log scale. Three dissociation pathways are identified. The strongest one leads to the formation of the (O^+^, O^+^) pair, while the other two, much weaker, produce (O^++^, O^+^) and (O^++^, O^++^).

Except for a few stable cationic states, most multi-charged Auger final states are dissociative. The dissociation pathways can be identified via a photoion-photoion coincidence map (PIPICO), as shown in Fig. 3. The Auger decays can take place either sequentially or non-sequentially, and the overall reaction processes can be written as:

1 $$\begin{array}{rcl}h\nu +{{\rm{O}}}_{2} & \to  & {{\rm{O}}}^{+}+{{\rm{O}}}^{+}+{e}_{ph}^{-}+{e}_{A}^{-}\end{array}$$

2 $$\begin{array}{rcl}\quad \quad \quad  & \to  & {{\rm{O}}}^{+}+{{\rm{O}}}^{++}+{e}_{ph}^{-}+2{e}_{A}^{-}\end{array}$$

3 $$\begin{array}{rcl}\quad \quad \quad  & \to  & {{\rm{O}}}^{++}+{{\rm{O}}}^{++}+{e}_{ph}^{-}+3{e}_{A}^{-}.\end{array}$$

Here, ${e}_{ph}^{-}$ and ${e}_{A}^{-}$ represent the photoelectron and the Auger electron(s), respectively.

Based on the above three equations the energy conservation should hold taking into account the photon energy, the photoelectron kinetic energy, the ion kinetic energies, the internal (electronic and vibrational) excitation energy of ions with respect to their ground state, and the Auger electron kinetic energies. It should be noted that using our electrostatic double toroidal analyzer we cannot measure simultaneously the photoelectron and the Auger electron(s). So, if more than one Auger electrons are produced, like in Eqs (2) and (3), there is no way to identify the Auger final states based on our experimental data. But, if only one Auger electron is produced, according to the Born-Haber cycle (see Fig. S1 of the Supplementary Material):

4 $$\begin{array}{rcl}E(h\nu ) & = & {E}_{K}({e}_{ph}^{-})+{E}_{K}({e}_{A}^{-})+{E}_{KER}\\  &  & +\sum _{i}\,({E}_{i}({{\rm{O}}}^{+}))+2{E}_{IT}({\rm{O}})+{E}_{D}({{\rm{O}}}_{2}).\end{array}$$

Here ${E}_{K}({e}_{ph}^{-})$ and ${E}_{K}({e}_{A}^{-})$ are the kinetic energy of the photoelectron and of the Auger electron, respectively. *E*_*KER*_ is the kinetic energy release, which is defined as the sum of the kinetic energies of all fragment ions. *E*_*i*_(O^+^) is the excitation energy of the *i*-th oxygen ion with respect to the ground state of an oxygen ion. *E*_*D*_ is the dissociation energy of the neutral O_2_ ground state. *E*_*IT*_(O) is the ionization threshold of the oxygen atom. The above formula can be rewritten as:

5 $${E}_{KER}={E}_{DIP}-{E}_{DL}$$

where the double ionization potential DIP ${E}_{DIP}=E(h\nu )-{E}_{K}({e}_{ph}^{-})-{E}_{K}({e}_{A}^{-})$ (the internal energy of the dication relative to the ground state neutral molecule), and the dissociation limit ${E}_{DL}={\sum }_{i}\,({E}_{i}({{\rm{O}}}^{+}))+2{E}_{IT}({\rm{O}})+{E}_{D}({{\rm{O}}}_{2})$. Because there are only a few excited states of O^+^ needed to be considered, the measured *KER* spectra reflect the Auger electron spectra indirectly.

Since the reaction processes (2) and (3) cannot be discussed in such a way because more than one Auger electrons are produced, one might question whether the discussion is affected by neglecting them. With an ionization threshold of 35 eV for O^+^, the kinetic energies of the Auger electrons from processes (2) and (3) will be at least 35 eV lower than those from processes (1). So only the O^+^/O^+^ ion pair can be produced in the range of Auger electron kinetic energies discussed.

**Figure 4:**
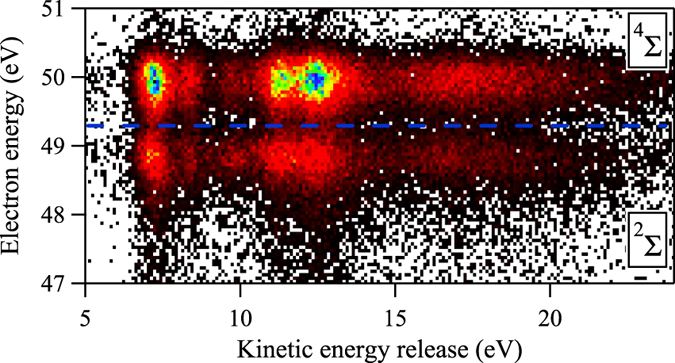
The correlation between the electron kinetic energy and the kinetic energy release of O^+^/O^+^ ion pair. The region above the blue dashed line contains decays from the $1{s}^{-1}({}^{4}{{\rm{\Sigma }}}^{-})$ intermediate state, and the one below from the $1{s}^{-1}({}^{2}{{\rm{\Sigma }}}^{-})$ state.

**Figure 5:**
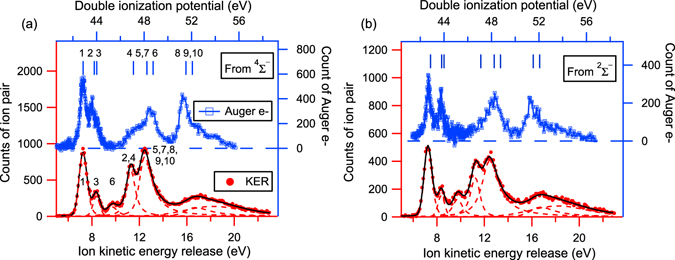
The correlation between the kinetic energy release of O^+^/O^+^ ion pairs and the kinetic energy of the Auger electrons. (**a**,**b**) Correspond to the intermediate states of ^4^Σ^−^ and ^2^Σ^−^, respectively. The Auger electron spectra are adapted from ref. 19. The labeling in (**a**) is adopted from Bao *et al*.^30^ using numbers from 1 to 10. The labeling in (**b**) is neglected as it is the same as that in (**a**). The definition of labeling can be found in column 1 and 2 of Table 3.

The correlation between the photoelectron kinetic energy and the *KER* is shown in Fig. 4, where several distinct peaks are resolved. Here we simply use a horizontal line at ${E}_{K}({e}_{ph}^{-})=49.3\,{\rm{eV}}$ to separate the 1*s* ^−1^ (^4^Σ^−^) from the 1*s* ^−1^ (^2^Σ^−^) intermediate ionic states. It should be noticed that due to the overlap between these two states and asymmetric profiles, the intensity of the 1*s* ^−1^ (^4^Σ^−^) state is underestimated by about 8%, and the intensity of the 1*s* ^−1^ (^2^Σ^−^) state is overestimated by about 15% by this simple separation. By projecting the events contained in the two zones individually on the horizontal axis, we get two *KER* spectra corresponding to these two intermediate states, individually. Then they are compared to the Auger electron spectra by Arion *et al*.^19^, as shown in Fig. 5. Here we adopt the labeling of the Auger final states from Bao *et al*.^30^, and the DIP of the Auger final states from Arion *et al*.^19^ to facilitate correlating the peaks in the *KER* spectra to those in the Auger electron spectra. The values of the DIP are increased by 0.26 eV to achieve the overall agreement of the energy levels. The two KER spectra seem similar at first sight, each spectrum being composed of five peaks and exhibiting one broad spectral feature on the higher energy side. However, the intensities of the peaks are quite different. To get the intensities, we fitted the *KER* spectra with the SPANCF curve-fitting package developed by Kukk as presented in refs 35 and 36. The positions of the five peaks are given in Table 2.

**Table 2:** Comparison of our least-square-fitting result with the intensity reported by Bao *et al*.^30^.

*KER* eV	Relat. Inten.				Contributing states
	^4^Σ^−^		^2^Σ^−^		
	Present	Bao	Present	Bao	
7.2	100	100	57^1^	41.8	1(*W* ^3^Δ_*u*_)
8.4	25^1^	45.0	15^1^	18.7	3(${B}^{^{\prime} 3}{{\rm{\Sigma }}}_{u}^{-}$)
9.8	27^2^	22.8	32^4^	11.1	6(^3^Π_*u*_)
11.2	104^8^	83.9	55^7^	36.6	2(*B* ^3^Π_*g*_) + 4(^3^Δ_*g*_)
12.4	199^22^	98.2	122^18^	32.7	5(^3^Π_*u*_) + 7(${}^{3}{\rm{\Sigma }}_{g}^{-}$)+
					8(^3^Π_*u*_) + 9(${}^{3}{\rm{\Sigma }}_{g}^{-}$)+
					10(${}^{3}{\rm{\Sigma }}_{g}^{-}$)

The Auger transition rates in each column are normalized to the first decay ^4^Σ^−^  → *W* ^3^Δ_*u*_. The errors are shown in square brackets.

During the KLL Auger process, one valence electron fills the core hole, while another one is emitted into the high-energy continuum. So, the configurations in which these two valence electrons form a singlet dominate the Auger decay processes. Since the oxygen molecule has a triplet ground state, the Auger final states are dominated by triplet states. Finally the molecular dications in triplet Auger final states dissociate into two, possibly excited, O^+^ ions.

To find the correlation between the molecular Auger final states and *KER* peaks, we display the double ionization potentials, the dissociation limits and corresponding possible *KER* s according to Eq. (5) in Table 3, together with the correlations found by Lundqvist *et al*.^32^ and Bao *et al*.^30^. It is quite clear that the first peak in the *KER* spectra is contributed from 1(*W* ^3^Δ_*u*_) states. Although Bao *et al*. suggested that the 2(*B* ^3^Π_*g*_) state would dissociate into ^4^S + ^2^D, with a peak appearing at *KER* = 8.1 eV, this peak is not observed by us. Concentrating on Auger electron spectrum, Bao *et al*. did not need to include the spin-orbit interaction induced dissociation into calculation. Instead, as suggested by Lundqvist *et al*.^32^ and later confirmed by Edvardsson *et al*.^37^, the 2(*B* ^3^Π_*g*_) dissociates through with the 1 ${}^{5}{\rm{\Sigma }}_{g}^{+}$ ^37^ into ^4^S + ^4^S with a *KER* peak at 11.4 eV, which is consistent with our observation. If the 3(${B}^{^{\prime} 3}{{\rm{\Sigma }}}_{u}^{-}$) dissociates adiabatically into ^4^S + ^2^P, a peak should appear at *KER* = 6.6 eV. But, instead, a peak at *KER* = 8.4 eV is observed close to estimated 8.3 eV in Table 3, which indicates that the 3(${B}^{^{\prime} 3}{{\rm{\Sigma }}}_{u}^{-}$) dissociates through a spin-orbit interaction with the 1 ^5^Π_*u*_^37^ into ^4^S + ^4^D. The peak at 9.8 eV can be contributed by either the 4(^3^Δ_*g*_) or the 6(^3^Π_*u*_), while the peak at 11.2 eV can be contributed by any of the 2(*B* ^3^Π_*g*_), 4(^3^Δ_*g*_), and 6(^3^Π_*u*_). Taking into account that the peak at 11.2 eV is much stronger than that at 9.8 eV, and that Bao *et al*. predicted that the decay probability to the 4(^3^Δ_*g*_) is much larger than that to the 6(^3^Π_*u*_), we suggest that the peak at 9.8 eV is contributed by the 6(^3^Π_*u*_), while the peak at 11.2 eV is contributed by the 2(*B* ^3^Π_*g*_), and the 4(^3^Δ_*g*_). The peak at *KER* = 12.4 eV is contributed by decays to 5(^3^Π_*u*_) + 7(${}^{3}{\rm{\Sigma }}_{g}^{-}$) + 8(^3^Π_*u*_) + 9(${}^{3}{\rm{\Sigma }}_{g}^{-}$) + 10(${}^{3}{\rm{\Sigma }}_{g}^{-}$) final states.

**Table 3:** The estimation of the *KER* from the double ionization potentials and the dissociation limits of the Auger final states.

Diss. lim.			^4^S + ^4^S 32.4	^4^S + ^2^D 35.7	^4^S + ^2^P 37.4	^2^D + ^2^D 39.0
Label						
1	*W* ^3^Δ_*u*_	42.9	10.5	**7**.**2** ^a,b^^,^^c^	5.5	3.9
2	*B* ^3^Π_*g*_	43.8	**11**.**4** ^a,c^	8.1^b^	6.4	4.8
3	${B}^{^{\prime} 3}{{\rm{\Sigma }}}_{u}^{-}$	44.0	11.6	**8**.**3** ^a,c^	6.6^b^	5.0
4	^3^Δ_*g*_	47.1	14.7	**11**.**4** ^a,b^	9.7^a^	8.1
5, 7	^3^Π_*u*_, ${}^{3}{\rm{\Sigma }}_{g}^{-}$	48.2	15.8	**12**.**5** ^a,b^	10.8	9.2
6	^3^Π_*u*_	48.8	16.4	13.1^c^	11.4^a,b^	**9**.**8** ^a^
8	^3^Π_*u*_	51.5	19.1	15.8	14.1	**12**.**5** ^a,b^
9, 10	${}^{3}{\rm{\Sigma }}_{g}^{-}$, ${}^{3}{\rm{\Sigma }}_{g}^{-}$	52.1	19.7	16.4	14.7	**13**.**1** ^a,b^

The first and second columns show the indexes and the assignments adopted from Bao *et al*.^30^, while the binding energies are adopted from Arion *et al*.^19^. Then the energies of the dissociation limits are calculated from the energy levels from NIST^44^. The numbers in bold font represent the assigned states. The energies are in eV.

^a^Observed now.

^b^Predicted by Bao *et al*.^30^.

^c^Observed by Lundqvist *et al*.^32^.

The profiles of peaks are important during the least-square fitting. Because the first three peaks are contributed by only one decay channel and their vibrational levels cannot be resolved with the present energy resolution, we assume that their energy position and shape do not change with respect to different intermediate states. As the next two peaks are contributed by several decays, their energy position and shape may depend on the corresponding intermediate state. The broad feature above 15 eV is fitted using two asymmetric peaks to take into account its effect on the former peaks. By normalizing to the decay ${}^{4,2}{\rm{\Sigma }}\to {W}^{3}{{\rm{\Delta }}}_{u}$, we obtain the relative intensities of the other Auger decays in Table 2. Bao *et al*.^30^ used a sloped line to represent the background in the spectrum, so they could have underestimated the intensities of Auger decays at lower electron kinetic energy where the Auger electron spectrum becomes apparently structure-less (corresponding to higher kinetic energy release here). From Table 2, it can be seen that for most Auger final states, the decay probability from ^4^Σ^−^ is much stronger than that from ^2^Σ^−^, but this rule is inverted at the third row that the Auger final state is 6(^3^Π_*u*_). This inversion of intensity is not observed before. Although Arion *et al*.^19^ and Bao *et al*.^30^ showed somewhat dependence of the intensities of the Auger decay transitions on the intermediate state, but the Auger line intensities from the ^4^Σ^−^ were always about two to three times those from the ^2^Σ^−^.

Now we would like to focus on the exceptionally small value of 1.2 of the $[1{s}^{-1}({}^{4}{{\rm{\Sigma }}}^{-})]/[1{s}^{-1}({}^{2}{{\rm{\Sigma }}}^{-})]$ intensity ratio in coincidence with ${{\rm{O}}}_{2}^{++}$ that is reduced to 55% of the non-coincidence value of 2.18. First, the TOF spectrum indicates that metastable ${O}_{2}^{++}$ Auger final state with a lifetime longer than 3.8 *μ*s exist. By double photoionization measurements, Eland revealed that below 46 eV the $X{}^{1}{\rm{\Sigma }}_{g}^{+}$, *W* ^3^Δ_*u*_, *B* ^3^Π_*g*_ and ${B}^{^{\prime} 3}{{\rm{\Sigma }}}_{u}^{-}$ electronic states of ${{\rm{O}}}_{2}^{++}$ are showing resolved vibrational progressions^38^. Therefore, the lifetimes of these states are longer than one hundred femtoseconds. Since it is very unlikely that bound dicationic states exist above 46 eV, they can be considered as the possible sources of stable ${{\rm{O}}}_{2}^{++}$. Second, we check whether the states can be reached by Auger decay and what is their lifetime. The $X{}^{1}{\rm{\Sigma }}_{g}^{+}$ state cannot be reached from ^2,4^Σ^−^ intermediate states, because in this case the continuum electron has to be of *σ* ^−^ symmetry and the symmetry of the one-electron wave function is completely violated. With a shallow potential well, the tunneling lifetime of the *W* ^3^Δ_*u*_ state is no longer than 0.7 ns^32^. As *B* ^3^Π_*g*_ state predissociates by spin-orbit coupling with the 1^5^Σ_*g*_ repulsive state, its lifetime is no longer than 3.5 ns^37^. ${B}^{^{\prime} 3}{{\rm{\Sigma }}}_{u}^{-}$ state can predissociate by spin-orbit coupling with the 1^5^Π_*u*_ repulsive state, ${B}^{^{\prime} 3}{{\rm{\Sigma }}}_{u}^{-}$ (*ν*″ = 0) having the longest lifetime that ranges from 0.1 to 0.8 *μ*s. This calculated lifetime can be increased to 10 *μ*s by slightly changing the potential curve within plausible errors^37^. Furthermore, ${B}^{^{\prime} 3}{{\rm{\Sigma }}}_{u}^{-}$ (*ν*″ = 0) was not observed in the Doppler free kinetic energy release spectrum by Lundqvist *et al*., and its lifetime should be longer than *μ*s. Finally, we suggest that the ${B}^{^{\prime} 3}{{\rm{\Sigma }}}_{u}^{-}$ (*ν*″ = 0) state is the candidate to be the source for the stable ${O}_{2}^{++}$ dication.

Since the ${B}^{^{\prime} 3}{{\rm{\Sigma }}}_{u}^{-}$ (*ν*″ = 0) state is metastable, we can evaluate the population of this vibrational sub-state by a Frank-Condon analysis from both, 1*s* ^−1^ (^4^Σ^−^) and 1*s* ^−1^ (^2^Σ^−^) intermediate states. According to Sorensen *et al*.^26^, 1*s* ^−1^ (^4^Σ^−^) is dominated by *ν*′ = 0 at the internuclear distance of 1.228 Å, and 1*s* ^−1^ (^2^Σ^−^) involves *ν*′ = 0, 1, 2 with the Franck-Condon factors of 0.571:0.309:0.097 at the internuclear distance of 1.269 Å. Lundqvist *et al*. pointed out that the potential of ${B}^{^{\prime} 3}{{\rm{\Sigma }}}_{u}^{-}$ has a minimum at 1.35 Å^32^. With the potential curves from them, we calculate the Franck-Condon factors for the decay $[1{s}^{-1}({}^{4}{{\rm{\Sigma }}}^{-})],[1{s}^{-1}({}^{2}{{\rm{\Sigma }}}^{-})]\to {B}^{^{\prime} 3}{{\rm{\Sigma }}}_{u}^{-}$, assuming Morse potentials, as shown in Table 4. Since the Franck-Condon factor of $({}^{4}{{\rm{\Sigma }}}^{-})({\nu }^{{\rm{^{\prime} }}}=0)\to {B}^{^{\prime} 3}{{\rm{\Sigma }}}_{u}^{-}({\nu }^{{\rm{^{\prime} }}{\rm{^{\prime} }}}=0)$ is much smaller than ${}^{2}{{\rm{\Sigma }}}^{-}({\nu }^{{\rm{^{\prime} }}}=0,1)\to {B}^{^{\prime} 3}{{\rm{\Sigma }}}_{u}^{-}({\nu }^{{\rm{^{\prime} }}{\rm{^{\prime} }}}=0)$, we can understand why the value of the $[1{s}^{-1}({}^{4}{{\rm{\Sigma }}}^{-})]/[1{s}^{-1}({}^{2}{{\rm{\Sigma }}}^{-})]$ ratio in coincidence with ${{\rm{O}}}_{2}^{++}$ is exceptionally smaller than the others.

**Table 4:** The Franck-Condon factors of the decay $[1{s}^{-1}({}^{4}{{\rm{\Sigma }}}^{-})],[1{s}^{-1}({}^{2}{{\rm{\Sigma }}}^{-})]\to ^{\prime} 3{{\rm{\Sigma }}}_{u}^{-}$ assuming Morse potentials.

*ν*′	${}^{{\bf{4}}}{{\boldsymbol{\Sigma }}}^{{\boldsymbol{-}}}{\boldsymbol{\to }}{{\boldsymbol{B}}}^{{\boldsymbol{^{\prime} }}3}{{\boldsymbol{\Sigma }}}_{{\boldsymbol{u}}}^{{\boldsymbol{-}}}$			${}^{{\bf{2}}}{{\boldsymbol{\Sigma }}}^{{\boldsymbol{-}}}{\boldsymbol{\to }}{{\boldsymbol{B}}}^{{\boldsymbol{^{\prime} }}3}{{\boldsymbol{\Sigma }}}_{{\boldsymbol{u}}}^{{\boldsymbol{-}}}$									Intensity ratio based on FC analysis [1*s* ^−1^ (^4^Σ^−^)]/[1*s* ^−1^(^2^Σ^−^)]		
	0			0			1			2					
*ν*″	0.954			0.571			0.309			0.097					
${r}_{{B}^{^{\prime} 3}{{\rm{\Sigma }}}_{u}^{-}}$(Å)	1.35	1.33	1.31	1.35	1.33	1.31	1.35	1.33	1.31	1.35	1.33	1.31	1.35	1.33	1.31
0	0.09	0.18	0.32	0.39	0.58	0.77	0.45	0.37	0.22	0.15	0.06	0.01	0.49	0.83	1.30
1	0.14	0.22	0.29	0.26	0.24	0.17	0.00	0.10	0.36	0.34	0.47	0.41			
2	0.15	0.18	0.18	0.14	0.10	0.04	0.07	0.18	0.24	0.10	0.00	0.08			
3	0.13	0.13	0.10	0.08	0.04	0.01	0.09	0.13	0.10	0.00	0.05	0.19			
4	0.10	0.09	0.05	0.05	0.02	0.01	0.08	0.08	0.04	0.01	0.08	0.13			
5	0.08	0.06	0.03	0.03	0.01	0.00	0.06	0.05	0.02	0.03	0.07	0.07			

Three values of the internuclear distance of ${B}^{^{\prime} 3}{{\rm{\Sigma }}}_{u}^{-}$ state are selected. The Franck-Condon factors for photoionization are adopted from Sorensen *et al*.^26^. The last column shows the expected intensity ratio of the two intermediate states $[1{s}^{-1}({}^{4}{{\rm{\Sigma }}}^{-})]$ and $[1{s}^{-1}({}^{2}{{\rm{\Sigma }}}^{-})]$ in coincidence with ${{\rm{O}}}_{2}^{++}$ assuming that they are populated with 2.18 ratio in the photoionization step.

However, with the Franck-Condon factors at ${r}_{{B}^{^{\prime} 3}{{\rm{\Sigma }}}_{u}^{-}}=1.35\,{\rm{\AA }}$, the expected intensity ratio of the two intermediate states $[1{s}^{-1}({}^{4}{{\rm{\Sigma }}}^{-})]$ and $[1{s}^{-1}({}^{2}{{\rm{\Sigma }}}^{-})]$ in coincidence with ${{\rm{O}}}_{2}^{++}$ is 0.49, which is by far below the measured value of 1.2. Previous studies^32, 37^, have shown that an error of 0.01–0.02 Å in the equilibrium distance is quite plausible. With this consideration in mind, we calculated the Franck-Condon factors with ${r}_{{B}^{^{\prime} 3}{{\rm{\Sigma }}}_{u}^{-}}$ shortened by 0.02 and 0.04 Å (see Table 4), respectively. The value of ratio become 0.83 and 1.3, respectively. A better agreement with the measured value of 1.2 is thus achieved.

Furthermore, from Fig. 1, it can be seen that the $1{s}^{-1}({}^{2}{{\rm{\Sigma }}}^{-})$ peak becomes narrower in coincidence with ${{\rm{O}}}_{2}^{++}$ compared to the non-coincident measurement, while the $1{s}^{-1}({}^{4}{{\rm{\Sigma }}}^{-})$ peak apparently does not change. From Table 4, the Franck-Condon factors ${}^{2}{{\rm{\Sigma }}}^{-}({\nu }^{{\rm{^{\prime} }}})\to {B}^{^{\prime} 3}{{\rm{\Sigma }}}_{u}^{-}({\nu }^{{\rm{^{\prime} }}{\rm{^{\prime} }}}=0)$ decrease dramatically as *ν*′ increases above 1, so the peak becomes narrower on its low kinetic energy side. This is in qualitative agreement with the measured spectrum.

## Conclusion

By measuring in coincidence the photoelectron and several ions, we are able to separate the Auger decay channels from the ^4^Σ^−^ and the ^2^Σ^−^ intermediate core-ionized states in molecular oxygen. Based on the correlation between the kinetic energy of the Auger electron and the kinetic energy release of the ion pair, we quantitatively obtain the intermediate state resolved Auger decay probabilities for single Auger electron emission. The Auger decay probability to a specific final state can dramatically depend on the intermediate state. Based on the channels leading to the production of ${{\rm{O}}}_{2}^{++}$, we identified that: *i*) The ${B}^{^{\prime} 3}{{\rm{\Sigma }}}_{u}^{-}(\nu ^{\prime\prime} =\mathrm{0)}$ state of the ${{\rm{O}}}_{2}^{++}$ dication is metastable and can be reached from both intermediate states; *ii*) The decay is vibrationally selective leading to a sharpening of the photoline when measured in coincidence with ${{\rm{O}}}_{2}^{++}$ dications.

We would finally like to compare the new experimental method proposed here with several other ones. Compared with ref. 19, the experimental statistics is greatly improved in our experiment, because we measured all ions emitted in the full solid angle of 4*π* steradian. Our result highlights a new way to explore the intermediate-state resolved Auger decay in terms of both energy resolution and statistical quality of the data. The method is generally applicable to any system and to any type of electronic decay, including Interatomic Coulombic Decay (ICD), for both stable and dissociative final states, as demonstrated here.

## Methods

**Figure 6:**
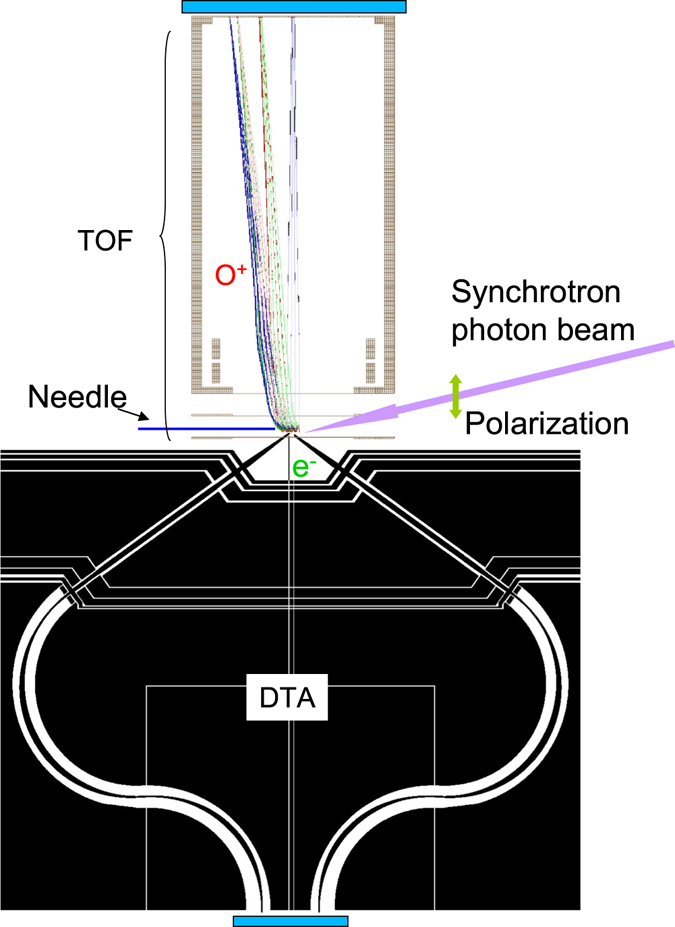
A schematic representation of the Auger electron-ion coincidence setup EPICEA^41^. Synchrotron radiation (SR) crosses an effusive beam of molecular oxygen from a non-magnetic metallic needle. Electrons are detected in a field-free region using an electrostatic Double Toroidal Analyzer (DTA), which collects electrons in the vicinity of the magic angle (54.7°) with respect to the symmetry axis^39^. The electron detection triggers a pulsed high voltage, which pushes ions into a 3-D focusing time-of-flight spectrometer. The ions with kinetic energies below 12 eV are collected. The kinetic energy and the emission angle of electrons, as well as the time of flight and the position of the ions, are recorded using two delay-line based position sensitive detectors.

The experiment was carried out at the PLEIADES beamline at the Synchrotron SOLEIL in France. As shown in Fig. 6, our electron-ion coincidence setup EPICEA consists of a double toroidal electron analyzer (DTA) and an ion time-of-flight (TOF) spectrometer. The detailed description of the instrumentation can be found in refs 39–42. The monochromatic photon beam was vertically polarized. It crosses the effusive gas beam generated using a gas needle with 200 *μm* inner diameter. The residual pressure in the reaction chamber was about 2 × 10^−8^ mbar before sample introduction, and it increased to about 2 × 10^−6^ mbar after sample introduction. Following photon absorption, the generated electrons travel through the retarding lens and the dispersive section of the analyzer^39^ and are further recorded by a delay-line position-sensitive detector DLD40 from Roentdek GmbH. The electron optics system allows collection of electrons emitted in the polar angle range of (54 ± 3°) with respect to the symmetry axis^40^, which corresponds to 4.2% of the full solid angle 4*π*. the double toroidal analyzer can only measure electrons with kinetic energy within a certain window, from 46.5 to 52.5 eV in present case. From the electron position (*x*_*e*_, *y*_*e*_), we determine the electron kinetic energy *E*_*e*_, the azimuthal angle *ϕ*_*e*_, and the momentum vector (*P*_*xe*_, *P*_*ye*_, *P*_*ze*_). The detection of the electron triggers a pulsed field (200 V between two meshes separated by 15.5 mm, rise time about 15 ns) of the ion spectrometer. The ions are detected by a hexagonal delay line detector with an active radius of 75 mm. Based on the time of flight and the impact position on the detector, (*t*_*i*_, *x*_*i*_, *y*_*i*_), the 3-D ion’s momentum (*P*_*xi*_, *P*_*yi*_, *P*_*zi*_) is calculated. During the measurements, the DTA was operated with an energy resolution of 0.4 eV and an angular resolution of 5°. The energy resolution for the ions was 0.5 eV, while the ion angular resolution was about 5°. When an ion pair is produced, the momentum conservation law is adopted in calculating the kinetic energy release of the ion pair in order to reduce the contribution from thermal motion and thus improve the energy resolution. In this case, the resolution of the ion kinetic energy is improved to about 0.2 eV. To reduce the contribution of random coincidence below 10%, the electron count rate was kept at less than 150 counts per second. The photon energy was 593.4 eV and its bandwidth was 50 meV. The random events were simulated using a random pulse generator at the repetition rate of 150 pulses/s and then their contribution was subtracted according to well-established procedures^43^.

## Electronic supplementary material

Supplementary Information
