# Supplementary material for: Disentangling Auger decays in O2 by photoelectron-ion coincidences
Source: Sci Rep. 2017 Jun 6;7:2898. doi: 10.1038/s41598-017-02875-x (PMC5460142; doi:10.1038/s41598-017-02875-x)
Supplement: Supplementary file 1 — Supplementary Information [file 41598_2017_2875_MOESM1_ESM.pdf]

Supplementary Information for

Disentangling Auger decays in O_2 by photoelectron-ion coincidences

Xiao-Jing Liu, Christophe Nicolas, Minna Patanen, and Catalin Miron

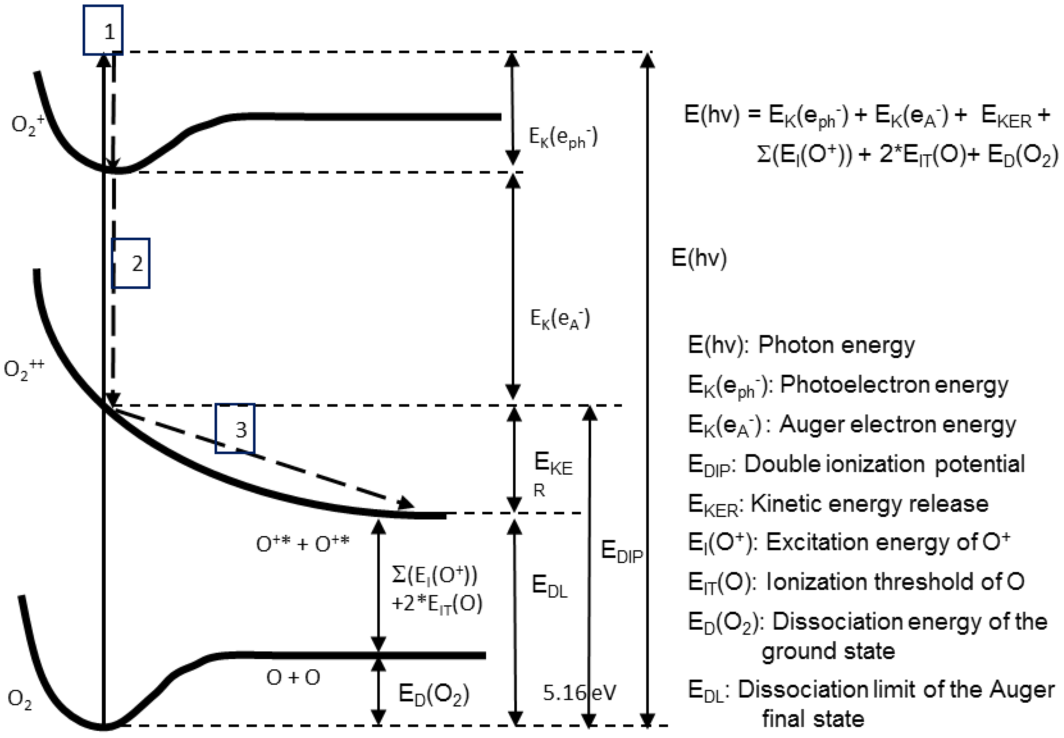

Figure s1. Born-Haber cycle during reactions:

1. photoionization, 2. Auger decay. 3 Dissociation
